# Medical Convention of Ohio

**Published:** 1838

**Authors:** 


### Art. IV.—The Medical Convention of Ohio.

Our last number was committed to the press before the meeting of this body, on the first Monday of January, and we could not, therefore, do more than lay its proceedings before our readers, in a supplement. We trust they have not been overlooked, nor even looked over merely, but read with attention. But, wherefore? We answer, because the topics on which that primary assembly of professional people occupied themseles, are of deep interest to our *caste*, and to society at large. The physicians of Ohio, not present on that interesting occasion, should read them, to learn what they are called upon to contribute towards the great works undertaken by the Convention; and our brethren in other states should study them, that they may be animated to *go and do* likewise. Nay, that they may do still better, but in the same way, that is, by spontaneous meetings, for mutual improvement of head and heart. If similar Conventions were held, in all the states of the Great Valley, its diversities of medical topography, climate, and diseases would soon be written down, and become the common property of the profession. Still further, a spirit of Inquiry would be awakened, in all its members, and a most beneficial impulse to improvement imparted. Finally, a love and practice of harmony would be promoted, where, it must be confessed, even the opposite too much prevails. The two Conventions of the physicians of Ohio, have demonstrated this; and should encourage the Faculty in the other western states, to resort to the same easy, pleasant and successful method, of raising themselves to a higher level, on the scale of industry, inquisitiveness and dignity. It should, however, be borne in mind, that mere meetings will not answer the purpose; and that they are in fact but the means of laying out business, and promoting harmony of vigorous action.

